# Associations of autonomous motivation and physical activity with mobile phone addiction and bedtime procrastination: a cross-sectional study

**DOI:** 10.1186/s40359-026-04157-6

**Published:** 2026-02-14

**Authors:** Jiawei Ren, Tengfei Zhu, Wencheng Liu, Jiale Li, Jianmin Jiang, Congchuan Ma

**Affiliations:** 1 School of Physical Education, Shanghai University of Sport, Shanghai, 200438 China; 2 Shanghai Jiading District Xinghui Primary School, Shanghai, 201803 China; 3 Shanghai Jiguang Senior High School, Shanghai, 200092 China

**Keywords:** Autonomous motivation, Physical activity, Mobile phone addiction, Bedtime procrastination, Chain mediation model, Multi-group analysis

## Abstract

**Background:**

Excessive pre-sleep mobile phone use fuels bedtime procrastination and sleep insufficiency, contributing to a self-reinforcing cycle of dependency. While physical activity benefits sleep health, the reasons for sustained engagement and the role of motivation quality in downstream outcomes remain understudied. This study integrates self-determination theory, compensatory internet use theory, the self-regulatory failure model, and the dual-system model to examine how autonomous motivation operating through physical activity is associated with mobile phone addiction and bedtime procrastination.

**Methods:**

In this cross-sectional study, 1,246 Chinese adults, including 389 university students (164 male, 225 female) and 857 working adults (524 male, 333 female), were recruited. The mean age of participants was 32.47 years (*SD* = 9.86). Validated scales were used to assess autonomous motivation (Behavioral Regulation in Exercise Questionnaire-3), physical activity (Physical Activity Rating Scale-3), mobile phone addiction (Mobile Phone Addiction Index), and bedtime procrastination (Bedtime Procrastination Scale). The data were analyzed using SPSS 23 and AMOS 24. Bootstrapped structural equation modeling was used to estimate the mediation effects and bias-corrected confidence intervals. Indirect effect magnitudes were quantified, and differences between standardized path coefficients (*Δβ*) were tested via confidence intervals and *p*-values. Multi-group SEM was used to evaluate model invariance across gender and occupational status.

**Results:**

The mediation analysis confirmed two significant pathways: a behavioral pathway controlling for autonomous motivation (physical activity → mobile phone addiction → bedtime procrastination, *β* = −0.006, *p* < .001, 1.6% of the total indirect effect) and an autonomous motivation-driven chain mediation pathway (autonomous motivation → physical activity → mobile phone addiction → bedtime procrastination, *β* = −0.071, *p* < .001, 17.8% of the total indirect effect). The autonomous motivation-driven pathway exerted a markedly stronger effect than the behavioral pathway did (*Δβ* = −0.065, *p* < .001), highlighting autonomous motivation’s amplifying role. Multi-group SEM confirmed full invariance across gender and occupation, supporting model robustness.

**Conclusions:**

This study provides initial evidence that autonomous motivation is associated with sustained physical activity and indirectly associated with lower levels of mobile phone addiction and bedtime procrastination. By contrasting motivation-driven and behavioral pathways within an integrative framework, the findings underscore the relevance of motivation-focused approaches for addressing technology-related sleep problems.

## Introduction

Excessive pre-sleep mobile phone use is a widespread issue that delays sleep and undermines well-being, forming a self-reinforcing cycle of mobile phone addiction and bedtime procrastination. Mobile phone addiction, a key manifestation of problematic use [7], involves psychological impairment and functional disruption in daily life. Prolonged screen exposure—including television, gaming, and social media—induces ocular strain and visual fatigue, potentially contributing to refractive errors such as myopia and astigmatism [17, 79]. Psychologically, individuals with mobile phone addiction exhibit elevated anxiety and depressive symptoms, especially during abstinence [22, 24], and tend to favor virtual over face-to-face interactions, increasing the risk of social isolation [1, 49].

Bedtime procrastination refers to voluntarily delaying sleep despite awareness of negative consequences [38], a behavior which can occur without external or physiological constraints [39]. As a distinct procrastination subtype, bedtime procrastination is closely linked to self-regulatory failure, including poor time management, reduced self-discipline, and electronic device dependency [69]. Pre-sleep mobile phone use—particularly of social media, videos-watching, and games—directly triggers intentional sleep postponement. Furthermore, with habitual device use, it amplifies procrastination severity, potentially disrupting circadian rhythms [40, 43, 51].

Hence, identifying effective strategies to mitigate these behaviors is crucial. Physical activity is known to support physiological and psychological well-being [8, 59], and to mitigate health-risk behaviors [36]. Importantly, emerging research demonstrates that physical activity is associated with lower levels of electronic device dependence, reduces excessive screen time and nonessential digital exposure [10, 73, 74], and is linked to sleep disorders [70, 80, 84]. However, its direct role in bedtime procrastination remains understudied. Evidence shows that physical activity improves sleep quality and alleviates insomnia [55]; however, findings on sleep timing are inconsistent, with acute exercise variably affecting sleep onset latency and wake time [82]. Beyond sleep, physical activity reduces general procrastination [84] and improves sleep health indicators [70], suggesting its promise for addressing maladaptive sleep-related patterns. Nevertheless, direct empirical tests linking physical activity to bedtime procrastination are scarce, and most studies rely on indirect physiological or behavioral proxies.

Although physical activity is known to reduce screen time and promote better sleep health, few studies have considered its broader influence on the reciprocal dynamics of mobile phone addiction and bedtime procrastination [15, 73, 83]. More critically, both lines of research largely overlook motivational processes—particularly why individuals engage in physical activity—even though such mechanisms are central to understanding their downstream effects. In addition, existing studies rarely explore the dynamic link between mobile phone addiction and bedtime procrastination, leaving their interplay insufficiently clarified.

Self-determination theory (SDT) provides a robust framework for understanding the motivational foundations of physical activity, distinguishing controlled motivation from autonomous motivation, including identified, integrated, and intrinsic regulations [21, 61]. Autonomous motivation is considered essential for sustaining health behaviors such as physical activity [20, 34, 71]. Beyond promoting adherence, autonomously motivated activity contributes to psychological well-being by fostering vitality, lower stress, and improved emotion regulation [6, 19]. These outcomes, in turn, may reduce susceptibility to maladaptive coping behaviors such as excessive mobile phone use and bedtime procrastination. However, motivational frameworks like SDT have rarely been integrated into models addressing technology-related behaviors and sleep regulation, leaving an important theoretical gap.

To address these gaps, this study introduces a novel theoretical integration to elucidate the underlying mechanisms involved. Grounded in SDT, we posit that autonomous motivation—the extent to which exercise is driven by personal value and interest—is the crucial catalyst for sustaining physical activity. We further propose that the benefits of such motivationally internalized physical activity may extend beyond physiological health to disrupt the cycle of bedtime procrastination through two interrelated pathways. To specify these mechanisms, we draw on three complementary theories.

First, compensatory internet use theory (CIUT) suggests that excessive phone use often serves as a maladaptive strategy to cope with unmet emotional needs [32]. In contrast, physical activity driven by autonomous motivation can fulfill basic psychological needs for autonomy, competence, and relatedness, which may reduce compensatory mobile phone use and subsequent addiction [24, 78].

**Fig. 1:**
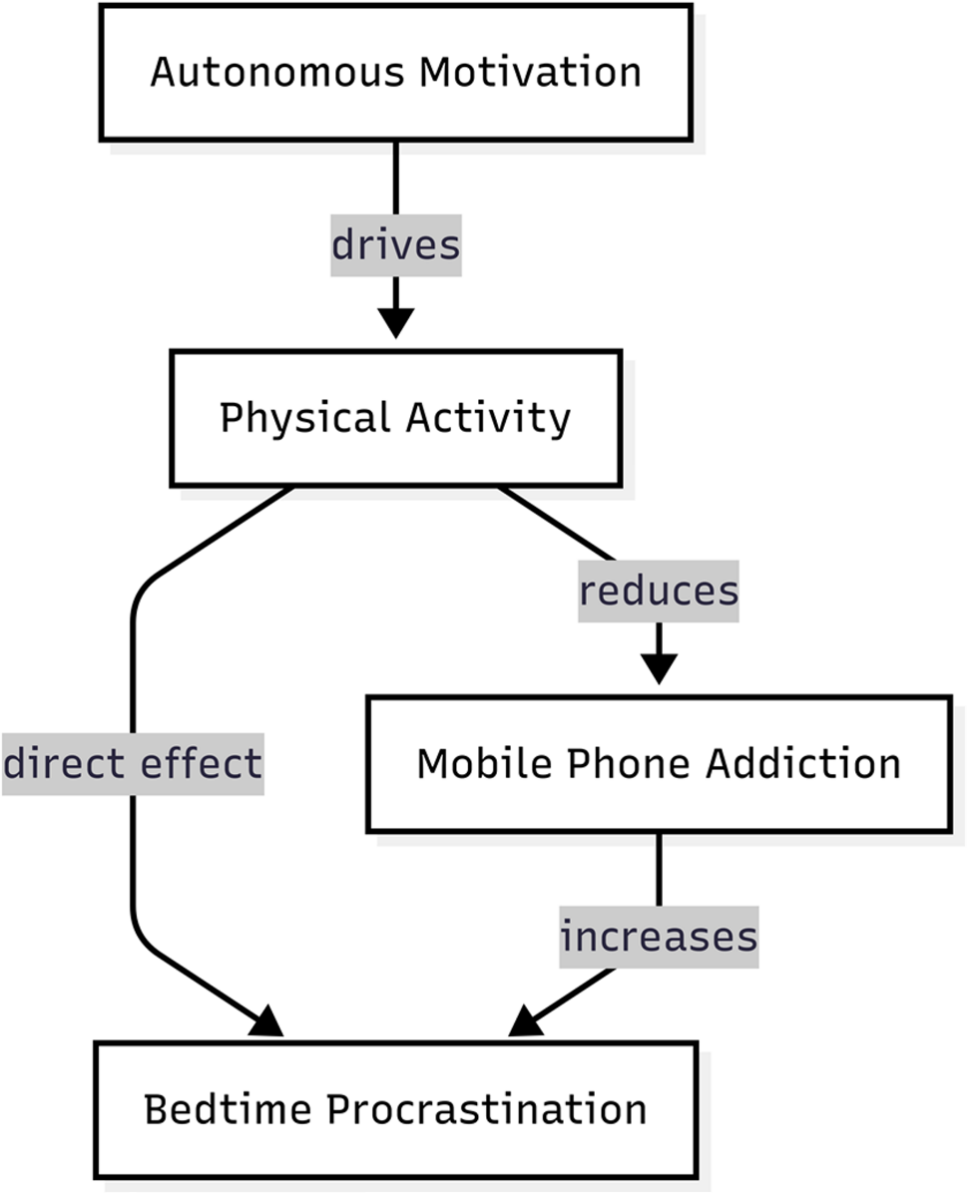
The Proposed Theoretical Model

Second, the self-regulatory failure (SRF) model and the dual-system model (DSM) emphasize that procrastination reflects impaired self-control [37, 39], wherein the impulsive system overrides reflective regulation [3, 4, 29]. Sustained physical activity may enhance self-regulatory resources, and when coupled with reduced phone addiction, this may help to restore evening cognitive balance and mitigate bedtime procrastination [25, 38, 50, 68]. Together, these perspectives provide a multidimensional rationale for the proposed framework (see Fig. 1).

As illustrated in Fig. 1, the integrated framework specifies distinct yet complementary theoretical roles across pathways. SDT delineates the link between autonomous motivation and higher levels of physical activity, CIUT accounts for the compensatory function of mobile phone use when psychological needs are unmet, while the SRF model and the DSM elucidate how depleted self-control and impulsive processes contribute to bedtime procrastination. Within this framework, autonomously motivated physical activity operates as a central buffering mechanism that mitigates both compensatory phone use and downstream self-regulatory failure.

Based on this integrated framework, the present study aims to test a holistic “motivation → behavior → mediator → outcome” pathway. Specifically, we examine a model in which autonomous motivation is linked to physical activity, which is in turn linked to lower mobile phone addiction, and subsequently to lower bedtime procrastination.

## Methods

### Participants and procedure

We conducted a cross-sectional study across six Chinese cities (Shanghai, Nanjing, Suzhou, Qingdao, Hangzhou, and Hefei) using convenience sampling. To ensure a diverse sample, data were collected at fitness centers and public community parks within each city. Eligible individuals were categorized into two groups: (1) university students and (2) working adults. Participants were approached while they were engaged in exercise or leisure activities. Out of the initial 1,300 distributed surveys, 1,246 valid responses were retained, yielding a response rate of 95.8%. The final sample consisted of 389 university students (164 male, 225 female) and 857 working adults (524 male, 333 female). Participants had a mean age of 32.47 years (SD = 9.86).

The study was reviewed and approved by the Shanghai University of Sport Scientific Research Ethics Committee and was conducted in accordance with the Declaration of Helsinki. Ethical approval was granted on December 9, 2024, prior to participant recruitment. Data collection occurred between February 5 and June 20, 2025. All participants provided written informed consent before participation. They were informed about the study’s purpose, procedures, the voluntary nature of participation, anonymity, confidentiality, and their right to withdraw at any time without penalty. Eligibility criteria required participants to be adults (aged 18 years or older), classified as either university students or working adults, and to have no severe cognitive or psychiatric impairments. For quality control, responses were excluded if the completion time was under three minutes, reverse-coded items were answered inconsistently, or stereotypical response patterns were detected (e.g., “1, 2, 3” or “3, 2, 1”). All data were anonymized and stored securely. The dataset will be retained by the corresponding author for five years before secure deletion.

### Measurement instruments

#### Behavioral regulation in exercise Questionnaire-3 (BREQ-3)

The Chinese version of the BREQ-3 [26] was used to assess autonomous motivation within the SDT framework. This scale has demonstrated reliability and validity across diverse Chinese populations [46]. The present study focused on three dimensions of autonomous motivation: identified regulation (ID), integrated regulation (IG), and intrinsic motivation (IM). These subscales together comprise a total of 12 items. This focus was theoretically driven, as autonomous motivation is central to sustaining exercise behavior through psychological need satisfaction, which is the core mechanism of our proposed model. In contrast, controlled motivation subtypes (e.g., external and introjected regulation) reflect externally regulated compliance and are less relevant to the internalized, self-regulatory processes under investigation. Including them might introduce theoretically inconsistent pathways and confound the interpretation of downstream effects. This operationalization aligns with prior research focusing on autonomous motivation as the primary explanatory construct in physical activity models [16, 18, 54]. The composite autonomous motivation score showed good internal consistency (Cronbach’s α = 0.823). Subscale reliabilities were also acceptable: α = 0.809 (ID), 0.819 (IG), and 0.780 (IM). All items were rated on a 5-point Likert scale (0 = not at all true; 4 = very true).

#### Physical activity rating Scale-3 (PARS-3)

The PARS-3 [44] was used to assess participants’ physical activity levels via three items measuring exercise intensity, duration, and frequency. Items used distinct 5-point scales—intensity and frequency scores ranged from 1 to 5, and duration scores ranging from 0 to 4. A total activity score was calculated as intensity × duration × frequency, yielding a maximum possible score of 100, with higher scores indicating greater activity. This scale has been widely used across various populations in China and has demonstrated stable reliability and validity [13, 81]. In the current study, it showed acceptable internal consistency (Cronbach’s *α* = 0.833).

#### Mobile phone addiction index (MPAI)

Mobile phone addiction was assessed using the revised Chinese version of the Mobile Phone Addiction Index [31], originally developed by Leung [41]. This 17-item instrument measures four subscales: inability to control craving, feeling anxious and lost, withdrawal/escape, and productivity loss. Items are rated on a 5-point Likert scale (1 = not at all; 5 = always). The reliability and validity of the scale have been reported in the Chinese population [12]. In this study, it demonstrated acceptable internal consistency overall (Cronbach’s *α* = 0.863) and for the subscales (*α* = 0.868, 0.837, 0.783, and 0.779, respectively).

#### Bedtime procrastination scale (BPS)

The revised Chinese BPS scale [47], originally developed by Kroese [38], was used to assess bedtime procrastination. This unidimensional scale has nine items that reflect behavioral frequency, time management, and sleep habits. Items are rated on a 5-point Likert scale (1 = never, 5 = always), with higher composite scores indicating greater severity of bedtime procrastination. The scale’s reliability and validity have been reported for Chinese populations [87]. In the present study, it showed excellent internal consistency (Cronbach’s *α* = 0.918).

### Statistical analysis

All analyses were conducted using SPSS 23.0 and AMOS 24.0. The integrated theoretical framework (incorporating SDT, CIUT, SRF, and DSM) was specified a priori before data collection. Structural equation modeling (SEM) was employed to test the hypothesized pathways derived from the integrated theoretical framework. Descriptive statistics and Pearson correlation analyses were performed first. Mediation effects were tested using bootstrapped SEM with 5,000 resamples and 95% bias-corrected confidence intervals (*CI*s). In addition to estimating the indirect effects, differences between standardized path coefficients (*Δβ*) were calculated to examine whether the strengths of different mediation pathways differed significantly; significance was assessed via *CI*s and *p*-values. Model fit was evaluated via multiple indices: absolute fit (*χ²*/df < 3, GFI/AGFI > 0.90), incremental fit (TLI > 0.90, NFI > 0.90, IFI > 0.90, CFI > 0.90), error approximation (RMSEA < 0.08), and standardized root mean square residual (SRMR < 0.05) [30, 76]. Mediation effects were interpreted based on whether the 95% *CI*s excluded zero. Common method bias was assessed using Harman’s single-factor test, which confirmed that the variance extracted by the first factor was less than 40% [56].

To examine whether gender and occupational status moderated the proposed mediation pathways, multi-group SEM was employed. The sample was divided by gender (male vs. female) and occupational status (student vs. working adult). A sequential constraint approach tested structural invariance. Model comparisons relied on chi-square difference tests (*Δχ²*), where a non-significant change (*p* >.05) suggested measurement invariance (i.e., no significant group differences in the path coefficients). For significant Δ*χ²* values, changes in incremental fit indices (ΔGFI, ΔAGFI, ΔCFI, and ΔRMSEA) were examined, with differences below 0.05 supporting invariance [35, 48, 65]. All statistical tests were considered significant at *p* <.05.

Additionally, to examine whether physical activity functioned as a full or partial mediator, alternative models including direct paths from autonomous motivation to mobile phone addiction and bedtime procrastination were tested.

## Results

### Common method bias

Common method bias was assessed using Harman’s single-factor test. The first factor explained 14.23% of the total variance, below the recommended threshold of 40% threshold, indicating negligible bias.

### Descriptive statistics and correlation analysis

Table 1 presents the means, standard deviations, skewness, kurtosis, and Pearson correlations among the core variables. All study variables met the criteria for approximate normality, with skewness values falling within ± 3 and kurtosis values within ± 10, which are considered acceptable thresholds [33, 52, 77]. Accordingly, Pearson’s correlation coefficients were calculated to examine associations among the variables. Key findings were as follows: First, autonomous motivation was positively correlated with physical activity (*r* =.551, *p* <.01). Second, physical activity was negatively correlated with both mobile phone addiction (*r* = −.164, *p* <.01) and bedtime procrastination (*r* = −.176, *p* <.01). Third, mobile phone addiction was positively correlated with bedtime procrastination (*r* =.214, *p* <.01). These correlational patterns align with the prerequisites for mediation analysis.

**Table 1:** Correlation matrix of study variables (*N* = 1,246)

Variable	M	SD	1	2	3	4	Skewness	Kurtosis
1. Autonomous motivation	2.003	0.827	-				−0.091	−0.680
2. Physical activity	28.21	30.627	0.551^**^	-			1.018	−0.150
3. Mobile phone addiction	2.978	0.782	0.23	− 0.164^**^	-		0.017	−0.544
4. Bedtime procrastination	3.023	1.087	0.008	− 0.176^**^	0.214^**^	-	0.012	−1.012

*Notes*. ^**^p <.01; *M* Mean, *SD* Standard deviation. Values represent composite or mean scores averaged across participants

### Multi-group analyses of autonomous motivation effects

The results of the multi-group analysis are presented in Tables 2 and 3. For both grouping variables (gender and occupational status), the unconstrained baseline models demonstrated acceptable fit to the data. As shown in Table 2, the occupational status model fit the data acceptably (*χ²*/df = 1.893, CFI = 0.970, GFI = 0.984, AGFI = 0.971, RMSEA = 0.027). The gender model also showed acceptable fit (*χ²*/df = 1.686, CFI = 0.977, GFI = 0.985, AGFI = 0.974, RMSEA = 0.023).

When equality constraints were progressively applied to the measurement weights, structural weights, structural covariances, and structural residuals, all constrained models maintained acceptable fit levels for both groups of variables. The *χ²*/df ratios ranged from 1.553 to 1.709, all comparative fit indices (CFI, GFI, AGFI) exceeded 0.90, and the RMSEA values remained below 0.08 across all the models.

Table 3 presents the invariance test results between nested models for both grouping variables. All chi-square difference tests were non-significant (all *p* >.05), and the changes in fit indices (ΔCFI, ΔGFI, ΔAGFI) remained within acceptable thresholds (all |Δvalue| < 0.05), whereas the ΔRMSEA values were less than 0.05. These results indicate that imposing cross-group constraints did not significantly degrade model fit. Thus, the structural model demonstrates full measurement and structural invariance across both gender and occupational status groups, suggesting that neither variable moderates the proposed mediation pathways.

**Table 2:** Multi-Group model fit indices by gender and occupational status

Model	χ²	DF	*p*	χ²/DF	CFI	GFI	AGFI	RMSEA
Occupational Status								
Unconstrained	2.758	7	0.906	1.893	0.970	0.984	0.971	0.027
Measurement weights	5.875	9	0.752	1.709	0.973	0.984	0.974	0.024
Structural weights	7.163	10	0.709	1.704	0.972	0.983	0.974	0.024
Structural covariances	7.327	11	0.772	1.697	0.972	0.983	0.974	0.024
Structural residuals	14.027	20	0.829	1.672	0.972	0.983	0.975	0.023
Measurement residuals	108.686	70	0.002	1.553	0.974	0.982	0.976	0.021
**Gender**								
Unconstrained	84.280	50	0.002	1.686	0.977	0.985	0.974	0.023
Measurement weights	89.009	57	0.004	1.562	0.978	0.985	0.976	0.021
Structural weights	91.120	59	0.005	1.544	0.978	0.984	0.976	0.021
Structural covariances	91.134	60	0.006	1.519	0.979	0.984	0.976	0.020
Structural residuals	92.461	61	0.006	1.516	0.978	0.984	0.976	0.020
Measurement residuals	105.041	70	0.004	1.501	0.976	0.982	0.977	0.020

Note. *χ²* chi-square, *DF* Degrees of freedom, *CFI* Comparative fit index, *GFI* Goodness-of-fit index, *AGFI* Adjusted goodness-of-fit index, *RMSEA* Root mean square error of approximation

**Table 3:** Invariance tests between nested models

Model	Δχ²	ΔDF	*p*	ΔCFI	ΔGFI	ΔAGFI	ΔRMSEA
Occupational Status							
Measurement weights	2.758	7	0.906	0.003	0.000	0.003	−0.003
Structural weights	5.875	9	0.752	0.002	−0.001	0.003	−0.003
Structural covariances	7.163	10	0.710	0.002	−0.001	0.003	−0.003
Structural residuals	7.327	11	0.772	0.002	−0.001	0.004	−0.004
Measurement residuals	14.027	20	0.829	0.004	−0.002	0.005	−0.006
**Gender**							
Measurement weights	4.729	7	0.693	0.001	0.000	0.002	−0.002
Structural weights	6.840	9	0.654	0.001	−0.001	0.002	−0.002
Structural covariances	6.854	10	0.739	0.002	−0.001	0.002	−0.003
Structural residuals	8.181	11	0.697	0.001	−0.001	0.002	−0.003
Measurement residuals	20.761	20	0.411	−0.001	−0.003	0.003	−0.003

Note. Δ*χ²* Change in chi-square, Δ*DF* Change in degrees of freedom, *p* Significance level of chi-square difference test, Δ*CFI* Change in comparative fit index, Δ*GFI* Change in goodness-of-fit index, Δ*AGFI* Change in adjusted goodness-of-fit index, Δ*RMSEA* Change in root mean square error of approximation

### Pathway analysis of autonomous motivation effects

#### Model fit and validation

**Fig. 2:**
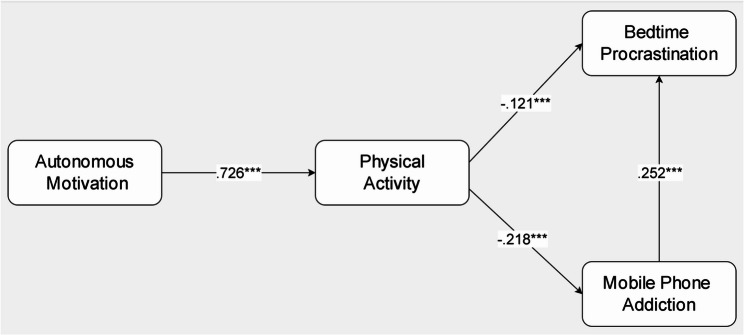
Standardized Path Coefficients of the Structural Equation Modeling. *Note.* ^***^p <.001

Given the establishment of measurement invariance, the full sample was used to test the proposed mediation model. As visualized in Fig. 2, the model integrates autonomous motivation, physical activity, mobile phone addiction, and bedtime procrastination within a cohesive structural model. Statistical evaluation confirmed acceptable fit across all indices: *χ²*/df = 2.526, GFI = 0.989, AGFI = 0.981, NFI = 0.958, IFI = 0.974, TLI = 0.963, CFI = 0.974, RMSEA = 0.035, SRMR = 0.040. All values exceeded established thresholds, substantiating the model’s capacity to capture the underlying relationships among the constructs.

#### Direct pathways: from autonomous motivation to behavioral outcomes

All direct pathways were statistically significant (all *p* <.001; Table 4). Specifically, autonomous motivation positively predicted physical activity (*β* = 0.726, *p* <.001). Physical activity was negatively associated with both mobile phone addiction (*β* = − 0.218, *p* <.001) and bedtime procrastination (*β* = − 0.121, *p* <.001). Mobile phone addiction, in turn, was positively associated with bedtime procrastination (*β* = 0.252, *p* <.001). These pathways, shown in Fig. 2, highlight the key association between autonomous motivation and physical activity within the model.

**Table 4:** Standardized direct effects from the SEM

Direct path	β	SE	*p*	
AM → PA	0.726	0.053	< 0.001	
PA → MPA	−0.218	0.037	< 0.001	
PA → BP	−0.121	0.029	< 0.001	
MPA → BP	0.252	0.04	< 0.001	

*Note. β* Standardized coefficient, *SE* Standard error. *AM *Autonomous motivation, *PA *Physical activity, *MPA *Mobile phone addiction, *BP *Bedtime procrastination

#### Indirect and chain mediation pathways

Bootstrap mediation analysis with 5,000 resamples revealed a significant total indirect effect of autonomous motivation on bedtime procrastination through physical activity and mobile phone addiction (*β* = −0.399, 95% CI [− 0.523, − 0.291], *p* <.001; see Table 5). Specifically, autonomous motivation was indirectly associated with reduced mobile phone addiction via physical activity (AM → PA → MPA; *β* = −0.166, 95% CI [− 0.232, − 0.109], *p* <.001) and with mitigated bedtime procrastination through physical activity (AM → PA → BP; *β* = −0.156, 95% CI [− 0.238, − 0.081], *p* <.001). Physical activity was further associated with decreased bedtime procrastination via its association with mobile phone addiction (PA → MPA → BP; *β* = −0.006, 95% CI [− 0.008, − 0.004], *p* <.001). Importantly, the full chain mediation pathway (AM → PA → MPA → BP) was also significant (*β* = −0.071, 95% CI [− 0.107, − 0.044], *p* <.001).

In terms of the proportion of total indirect effects, AM → PA → MPA accounted for 41.5%, AM → PA → BP explained 39.1%, PA → MPA → BP contributed 1.6%, and the full chain mediation pathway (AM → PA → MPA → BP) represented 17.8%. Contrast analyses revealed no significant difference between the AM → PA → MPA and AM → PA → BP (*Δβ* = 0.009, 95% CI [− 0.089, 0.111], *p* =.845). However, the chain mediation pathway (AM → PA → MPA → BP) was significantly stronger than the pathway excluding autonomous motivation (*Δβ* = −0.065, 95% CI [− 0.101, − 0.038], *p* <.001).

**Table 5:** *Mediating effects with bootstrapped confidence intervals (5*,*000 iterations)*

	Effect type	Mediation path	β	*p*	95% CI [lower–upper]	
Indirect effects (standardized β)						
	Total Effect	—	− 0.399	< 0.001	− 0.523	− 0.291
	Indirect 1	AM → PA → MPA	− 0.166	< 0.001	− 0.232	− 0.109
	Indirect 2	AM → PA → BP	− 0.156	< 0.001	− 0.238	− 0.081
	Indirect 3	PA → MPA → BP	− 0.006	< 0.001	− 0.008	− 0.004
	Indirect 4	AM → PA → MPA → BP	− 0.071	< 0.001	− 0.107	− 0.044
Proportion of the total indirect effect						
	Indirect 1	AM → PA → MPA	0.415	< 0.001	0.311	0.528
	Indirect 2	AM → PA → BP	0.391	< 0.001	0.23	0.535
	Indirect 3	PA → MPA → BP	0.016	< 0.001	0.012	0.019
	Indirect 4	AM → PA → MPA → BP	0.178	< 0.001	0.12	0.252
Contrasts of indirect effects (*Δβ*)						
	*Δβ1*	Indirect 1 vs. Indirect 2	0.009	0.845	− 0.089	0.111
	*Δβ2*	Indirect 3 vs. Indirect 4	− 0.065	< 0.001	− 0.101	− 0.038

Note. *β* Standardized indirect effect, *Proportion* Proportion of the total indirect effect explained by each pathway, *Δβ* Difference between two indirect effects, *CI* Confidence interval, *AM *Autonomous motivation, *PA *Physical activity, *MPA *Mobile phone addiction, *BP *Bedtime procrastination, AM → PA → MPA → BP= autonomous motivation–driven pathway; PA → MPA → BP= behavioral pathway (controlling for AM)

### Alternative mdel including direct paths from autonomous motivationo

**Fig. 3:**
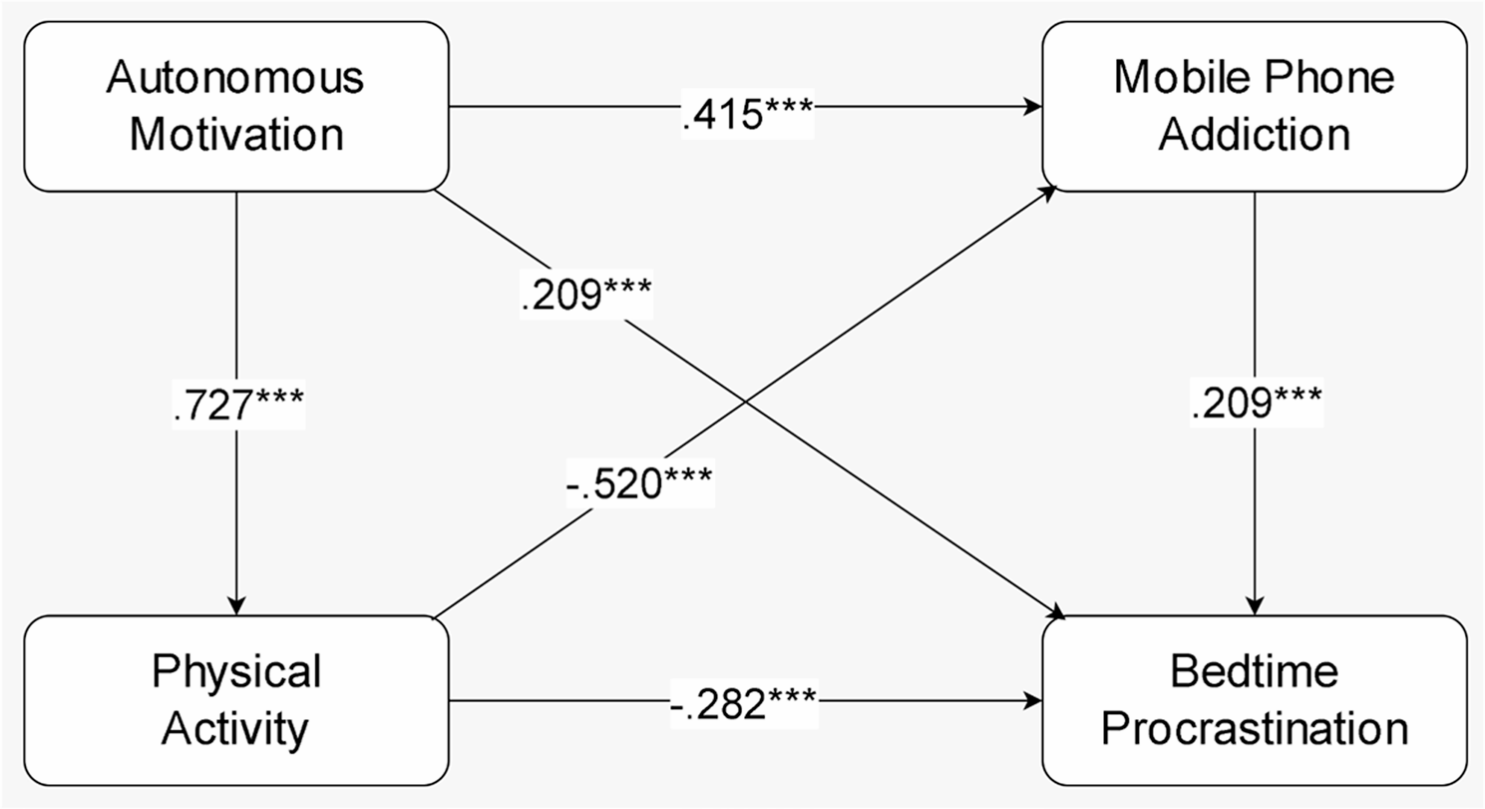
Standardized Path Coefficients for the Alternative Model. Note. ^***^p <.001

To examine whether physical activity functioned as a full or partial mediator, we tested an alternative structural model that included direct paths from autonomous motivation to mobile phone addiction and bedtime procrastination (AM→MPA, AM→BP; see Fig. 3). This alternative model demonstrated acceptable overall fit to the data: *χ²*/df = 1.049, GFI = 0.996, AGFI = 0.992, TLI = 0.999, NFI = 0.984, IFI = 0.999, CFI = 0.999, RMSEA = 0.006, SRMR = 0.016.

As shown in Table 6, within this alternative model, autonomous motivation remained a strong positive predictor of physical activity (*β* = 0.727, *p* <.001). Physical activity was negatively associated with mobile phone addiction (*β* = − 0.520, *p* <.001) and bedtime procrastination (*β* = − 0.282, *p* <.001). Mobile phone addiction was positively associated with bedtime procrastination (*β* = 0.209, *p* <.001).

Although the added direct paths were statistically significant, their positive direction was inconsistent with the theoretical rationale. Specifically, the paths from autonomous motivation to mobile phone addiction (*β* = 0.415, *p* <.001) and to bedtime procrastination (*β* = 0.209, *p* <.001) were positive. Given the theoretical inconsistency of these positive direct effects, the original mediation model (without these direct paths) is supported and retained for interpretation.

**Table 6:** Standardized direct path coefficients for the alternative model

Direct path	β	SE	*p*
AM → PA	0.727	0.053	< 0.001
PA → MPA	−0.52	0.078	< 0.001
AM → MPA	0.415	0.095	< 0.001
PA → BP	−0.282	0.063	< 0.001
MPA → BP	0.209	0.042	< 0.001
AM → BP	0.209	0.126	< 0.001

*Note. β* Standardized coefficient, *SE* Standard error. *AM *Autonomous motivation, *PA *Physical activity, *MPA *Mobile phone addiction, *BP *Bedtime procrastination

## Discussion

### Theoretical contribution: unveiling the motivational core of behavior change

This study provides initial evidence for a theoretically grounded association whereby autonomous motivation–driven physical activity is linked to lower levels of mobile phone addiction and bedtime procrastination. Moving beyond prior work that primarily emphasized the direct correlates of general physical activity [5, 28], our findings underscore motivational quality as a central explanatory component within this behavioral chain. By integrating SDT with CIUT, SRF, and DSM, we propose a multi-theoretical framework. This framework posits that internalized motivation is associated with higher levels of physical activity, which in turn is linked to more adaptive self-regulatory functioning and less problematic technology-related and sleep-related behaviors. In this sense, autonomous motivation emerges as a theoretically meaningful upstream construct that may help account for why physical activity shows stronger associations with downstream behavioral outcomes, thereby extending and refining existing research.

### Reciprocal dynamics of the variables and direct path findings

The direct path estimates, considered alongside the correlational patterns, highlight robust interrelationships among the core variables. The results indicate that autonomous motivation was positively associated with physical activity (*r* =.551, *p* <.01; *β* = 0.726, *p* <.001). Physical activity, in turn, was negatively associated with mobile phone addiction (*r* = −.164, *p* <.01; *β* = −0.218, *p* <.001) and bedtime procrastination (*r* = −.176, *p* <.01; *β* = −0.121, *p* <.001). Additionally, mobile phone addiction showed a positive association with bedtime procrastination (*r* =.214, *p* <.01; *β* = 0.252, *p* <.001).

Taken together, this pattern suggests a set of closely interrelated behavioral tendencies: higher levels of mobile phone addiction co-occur with lower physical activity and greater bedtime procrastination, whereas greater bedtime procrastination is also associated with higher mobile phone addiction and lower physical activity. These associations are consistent with prior evidence indicating that excessive mobile phone use is linked to reduced engagement in physical activity through temporal displacement and compromised self-regulatory capacity [14, 86]. From the perspective of CIUT, maladaptive mobile phone use may function as a short-term coping strategy, which is associated with heightened cognitive arousal and delayed sleep timing [42]. Although the present cross-sectional design does not permit causal or temporal inferences, the observed pattern of associations aligns with a potentially reinforcing behavioral configuration in which lower physical activity, higher mobile phone addiction, and greater bedtime procrastination tend to co-occur [31, 75]. Within the SRF and CIUT frameworks, this configuration illustrates how technology-related behaviors may become habitual yet ineffective self-regulatory strategies when health-promoting routines such as regular physical activity are less salient [51].

### Generalizability of the model across demographics

Based on the multi-group analysis results, the structural invariance across gender and occupational status groups indicates that the mediating pathways show similar patterns regardless of these demographic factors. This indicates that the relationship between mobile phone addiction and bedtime procrastination is broadly consistent across demographic groups, rather than group-specific. Such findings align with previous research indicating that excessive mobile phone use leads to delayed bedtime across diverse populations [11, 83], supporting the notion that technology-related sleep disruption represents a broadly shared behavioral phenomenon in contemporary digital contexts. Similarly, the negative association between physical activity and mobile phone addiction observed in this study was comparable across gender and occupational groups, indicating that these relationships are not confined to specific demographic subgroups [15]. Importantly, the absence of significant moderation effects should be interpreted cautiously. While the present findings suggest structural similarity across groups, they do not imply that intervention strategies would be equally effective across all populations. Rather, the results indicate that the core associations linking autonomous motivation, physical activity, mobile phone addiction, and bedtime procrastination may be broadly relevant across demographic segments. Future research employing longitudinal or intervention designs is needed to determine whether these associations translate into comparable behavioral change processes across different groups. Additionally, further studies should examine whether other individual or cultural factors may condition or bound the generalizability of these relationships ([11]; Ryan & Deci, (63) b).

The establishment of full measurement and structural invariance across gender and occupational status is a noteworthy finding that extends beyond a mere statistical check. It suggests that the theoretical linkages posited in our integrated model—whereby autonomous motivation supports physical activity, which in turn is associated with lower levels of mobile phone addiction and, consequently, bedtime procrastination—represent a robust psychological and behavioral sequence that operates similarly across these fundamental demographic divides. This invariance strengthens the theoretical claim that the core mechanisms drawn from SDT, CIUT, and SRF/DSM—the role of need satisfaction, compensatory behavior, and self-regulatory resource dynamics—may reflect fundamental processes underlying technology-related sleep issues, rather than artifacts of group-specific contexts.

From a theoretical perspective, such invariance may be explained by the fact that the core mechanisms underlying the model are rooted in broadly universal psychological processes. Basic psychological needs posited by SDT are assumed to be inherent and essential across social groups (Ryan & Deci, [63] b), the compensatory use of mobile phone in response to unmet needs reflects a general functional tendency rather than a context-specific habit [32], and self-regulatory resource limitations represent a common constraint on human behavior [4, 25]. Accordingly, the observed invariance lends theoretical support to the view that these mechanisms operate at a general psychological level, rather than being contingent on specific demographic contexts.

From a methodological standpoint, demonstrating both measurement and structural invariance is critical for establishing the validity of cross-group comparisons in structural equation modeling. Measurement invariance indicates that the latent constructs are conceptualized and measured equivalently across groups, whereas structural invariance suggests that the relationships among these constructs operate in a comparable manner [33, 72]. In the present study, the establishment of full invariance strengthens the credibility of the proposed mediation structure by indicating that the estimated pathways are not artifacts of sample composition or group-specific measurement properties, but reflect stable associative patterns across gender and occupational status. Importantly, structural invariance does not imply that intervention effects would be identical across groups; rather, it indicates that the underlying motivational–behavioral associations are expressed in a consistent manner across gender and occupational status, providing a methodological basis for future subgroup-specific, longitudinal, or experimental investigations.

### Mechanism-Oriented pathway interpretation

The empirical analyses demonstrated that autonomous motivation was indirectly associated with lower levels of mobile phone addiction and bedtime procrastination through the mediating role of physical activity. Specifically, autonomous motivation was linked to reduced mobile phone addiction (indirect effect via physical activity: *β* = −0.166, 95% CI [− 0.232, − 0.109], *p* <.001) and lower bedtime procrastination (*β* = −0.156, 95% CI [− 0.238, − 0.081], *p* <.001). These findings suggest that when physical activity is sustained by internalized motivation, it may function as a behavioral conduit associated with improvements in excessive mobile phone use and sleep-related behaviors, rather than operating as an isolated health practice. The nonsignificant difference between these two indirect pathways (*Δβ* = 0.009, *p* =.845) indicates that autonomously motivated physical activity exhibits comparable associative strength across these behavioral outcomes, consistent with SDT’s proposition that internalized motivation supports generalized self-regulatory capacity rather than producing domain-specific effects [9, 34, 60].

Additionally, mobile phone addiction partially mediated the relationship between physical activity and bedtime procrastination (*β* = −0.006, 95% CI [− 0.008, − 0.004], *p* <.001). This suggests that physical activity may be associated with lower bedtime procrastination partly through displacement of maladaptive phone use even in the absence of autonomous motivation. However, the relatively small magnitude of this indirect effect suggests that behavioral engagement alone represents a limited self-regulatory pathway, underscoring the importance of motivational quality for more substantial behavioral associations [51, 53].

The full chained mediation pathway—from autonomous motivation to physical activity, then to mobile phone addiction, and finally to bedtime procrastination—was also significant (*β* = −0.071, 95% CI [− 0.107, − 0.044], *p* <.001), accounting for 17.8% of the total effect. In relative terms, this indirect pathway was substantially larger than the behavioral pathway through mobile phone addiction alone while controlling for autonomous motivation (1.6% of the total effect), highlighting the amplifying role of autonomous motivation. This pattern is consistent with prior evidence indicating that intrinsically regulated physical activity is associated with broader psychosocial benefits [54, 64]. While the observed indirect effects—particularly the full chain pathway from autonomous motivation through physical activity and mobile phone addiction to bedtime procrastination—were modest in absolute magnitude, this pattern is theoretically expected and substantively meaningful. Chain mediation models inherently involve the transmission of effects across multiple psychological and behavioral stages, which typically results in attenuated effect sizes at distal outcomes—a pattern observed in similar studies across the social sciences [2, 85]. In this context, autonomous motivation represents a distal psychological construct whose influence must be translated into observable behavioral engagement before affecting complex self-regulatory outcomes such as bedtime procrastination. Moreover, bedtime procrastination is a multifactorial behavior influenced by diverse contextual, occupational, and psychosocial factors [14, 15], making it unlikely that any single motivational or behavioral pathway would exert a large standalone association. From a behavioral health perspective, these small but reliable indirect effects likely reflect incremental processes that may accumulate across repeated daily decisions, positioning autonomous motivation as a foundational psychological resource within broader, multi-component behavioral frameworks rather than as an isolated determinant [39].

The significantly stronger effect of the autonomous motivation-driven chain pathway (AM → PA → MPA → BP) compared to the behavioral pathway (PA → MPA → BP) underscores a critical theoretical distinction. The behavioral pathway primarily reflects a temporal displacement or resource competition mechanism; engaging in physical activity reduces the available time and opportunity for excessive mobile phone use. In contrast, the autonomous motivation pathway, grounded in SDT, operates through a more profound psychological mechanism. Autonomously motivated physical activity is inherently linked to greater satisfaction of basic psychological needs [20, 21]. This need satisfaction reduces the propensity to turn to the mobile phone as a maladaptive compensatory strategy for coping with unmet needs [32]. Furthermore, activities driven by internalized motivation, such as autonomous motivation, are less depleting and may be less depleting and potentially supportive of self-regulatory resources, thereby strengthening an individual’s capacity for reflective rather than impulsive decision-making in the evening [4, 25]. Thus, physical activity embedded within an autonomous motivational context is more likely to exert broader spillover effects on maladaptive mobile phone using behaviors and sleep-related self-regulatory outcomes. This theoretical distinction helps explain why the motivation-driven chain pathway demonstrated a substantially stronger indirect association than the behavioral pathway alone.

It is noteworthy that the bivariate correlation between autonomous motivation and bedtime procrastination was non-significant (*r* =.008, *p* >.05), whereas the indirect association through physical activity and mobile phone addiction was statistically significant. This pattern is theoretically and statistically plausible. Prior methodological work has demonstrated that significant indirect effects can exist even in the absence of a significant zero-order correlation, particularly when the relationship between variables operates entirely through mediating mechanisms rather than direct pathways [27, 57]. In the present study, autonomous motivation represents a distal psychological factor that does not directly influence bedtime procrastination, but instead exerts its effect by shaping health-related behaviors (physical activity), which subsequently alter maladaptive technology use and sleep-related self-regulation. This full mediation structure explains why the total association emerges only when intermediate processes are considered.

Collectively, these findings indicate that physical activity is associated with bedtime procrastination through both behavioral substitution processes—displacing maladaptive mobile phone use—and physiological regulation related to fatigue and circadian alignment [59, 74]. Importantly, the stronger associations observed in the autonomously motivated pathway underscore that it is not merely engagement in physical activity, but the quality of motivation underlying that engagement, that is linked to more favorable sleep-related outcomes [11, 58, 83]. Accordingly, the theoretically specified behavior-mediated model provides a conceptually coherent and parsimonious account of the observed associations and is retained as the primary explanatory framework.

### Model specification and theoretical justification of mediation structure

To further evaluate whether physical activity functioned as a full or partial mediator, an alternative model including direct paths from autonomous motivation to mobile phone addiction and bedtime procrastination was tested. The emergence of statistically significant but theoretically inconsistent positive direct paths from autonomous motivation to mobile phone addiction and bedtime procrastination in the alternative model warrants careful interpretation. In cross-sectional data, such anomalous associations may reflect incomplete model specification rather than genuine causal effects.

Methodologically, prior research indicates that excluding a key behavioral mediator may result in distorted or counterintuitive parameter estimates in mediation models ([33]; Wen, 2004). When physical activity is not explicitly modeled, autonomous motivation may be treated as if it could exert effects on downstream outcomes in isolation. However, autonomous motivation is a distal motivational construct that requires behavioral enactment to produce adaptive consequences [20]. Consistent with a well-documented intention–behavior gap, motivational orientations do not necessarily translate into corresponding behavioral engagement [66]. In the absence of physical activity, a condition of “motivation without action” may emerge, in which motivational resources fail to be converted into sustained behavior. Within this context, unfulfilled motivational intentions may be accompanied by unmet psychological needs, which, according to CIUT and SRF perspectives, are psychological states that increase vulnerability to maladaptive coping behaviors, including excessive mobile phone use and bedtime procrastination [32]. Thus, the positive direct paths in the alternative model likely reflect the consequences of omitting the core behavioral process, not beneficial motivational effects.

By contrast, the hypothesized mediation model explicitly positions physical activity as the critical behavioral conduit through which autonomous motivation is translated into adaptive self-regulatory outcomes. When physical activity is properly specified as a mediator, the theoretically inconsistent direct associations are attenuated or absorbed into the indirect pathway, thereby restoring conceptual coherence between motivation, behavior, and downstream outcomes. Accordingly, these findings provide support for the specification of physical activity as an indispensable mediator in the motivational–behavioral chain.

### Theoretical integration: an SDT–CIUT–SRF/DSM framework for disrupting behavioral cycles

**Fig. 4:**
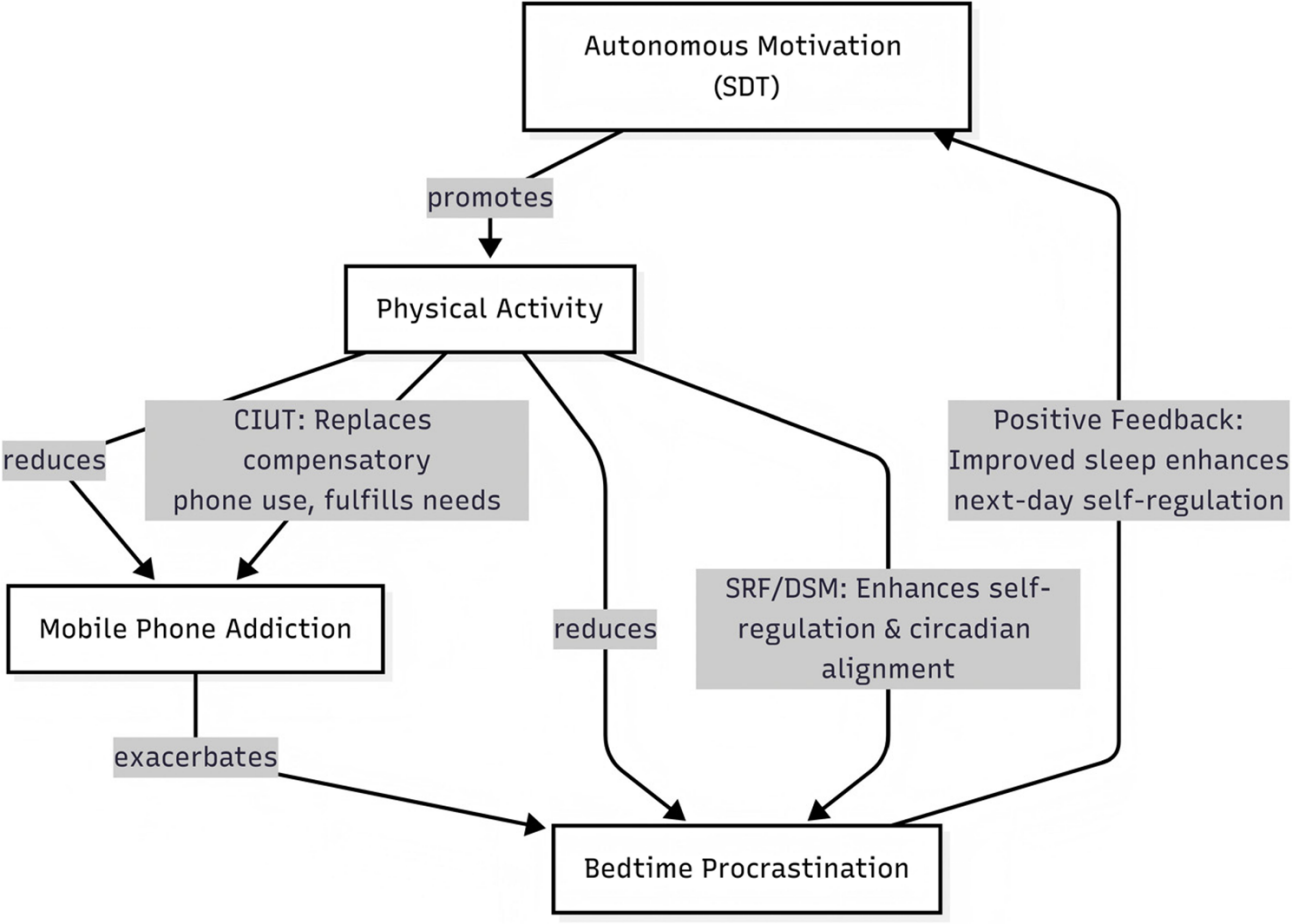
Tripartite model of motivation-driven physical activity and outcomes

Beyond the statistical mediation patterns reported above, the present findings can be conceptually interpreted within an integrative SDT–CIUT–SRF/DSM framework (see Fig. 4). This framework provides a coherent account of how autonomous motivation–driven physical activity is associated with reduced mobile phone addiction and bedtime procrastination.

From an SDT perspective, autonomous motivation reflects the internalization of physical activity as a personally valued behavior, which is linked to the satisfaction of basic psychological needs, particularly autonomy (Ryan & Deci, [62] a). Such internalization is theorized to support sustained engagement in physical activity, serving as the necessary behavioral foundation for downstream self-regulatory processes.

The CIUT further contextualizes these associations by highlighting the compensatory role of mobile phone use in response to unmet psychological needs [32]. Physical activity sustained by autonomous motivation may function as a health-promoting alternative that fulfills similar needs while occupying time and attentional resources, thereby being associated with lower reliance on compensatory mobile phone use [23].

The SRF and DSM frameworks offer additional insight into bedtime procrastination as a self-regulatory phenomenon characterized by diminished reflective control and heightened impulsive tendencies in the evening [4, 39]. Within this context, regular physical activity has been associated with improved circadian alignment and psychological resource restoration, which may support more effective regulation of technology use and sleep-related decisions [29, 45, 68].

Collectively, this integrative framework delineates a coherent trajectory from distal motivational orientations to behavioral engagement and subsequent sleep-related outcomes. By positioning autonomous motivation as the core psychological correlate and physical activity and mobile phone addiction as key behavioral mediators—constructs often examined separately in prior research—this model clarifies how motivation-related processes may be linked to sleep timing through multiple intermediate mechanisms. Moreover, reduced mobile phone addiction and improved sleep may reciprocally support next-day self-regulation, forming a potential positive feedback loop that reinforces sustained engagement in physical activity [67]. While these processes are conceptual in nature, they provide a theoretically grounded interpretation of the observed associations and highlight avenues for future longitudinal and experimental investigation.

#### Limitations

Several limitations should be acknowledged. First, although the present model was theoretically specified, the cross-sectional design precludes causal inferences about the direction and temporal sequence of the proposed pathways. Longitudinal or experimental studies are therefore needed to verify causal mechanisms and developmental dynamics. Second, the sample was drawn from urban areas in Eastern China and recruited primarily from public venues such as fitness centers and parks. This may have resulted in a cohort with relatively higher levels of physical activity or health-related motivation. This sampling context may constrain the generalizability of the findings to rural populations or individuals with lower baseline engagement in physical activity.

Third, key demographic moderators such as age and income were not explicitly examined and may influence the strength or form of the observed associations. Future studies should test whether these relationships vary across demographic subgroups and cultural contexts.

Fourth, all variables were assessed via self-report measures, and the absence of objective physiological indicators limits direct validation of the proposed sleep-related mechanisms.

Finally, the present study focused exclusively on autonomous motivation. Although theoretically justified, future research should explicitly compare autonomous and controlled motivation profiles to disentangle their distinct roles in technology-related behaviors and sleep outcomes.

## Conclusion

This study provides empirical evidence highlighting the important roles of autonomous motivation and physical activity in relation to mobile phone addiction and bedtime procrastination. The findings suggest that physical activity sustained by autonomous motivation is associated with lower mobile phone addiction and bedtime procrastination through two interrelated mechanisms: temporal substitution (displacing phone use) and physiological regulation related to cognitive resource restoration.

Notably, multi-group analyses revealed no significant differences in these pathways across occupational status or gender, indicating that the observed associations are robust across key demographic groups. The internalization of motivation may support sustained engagement in physical activity, which is linked to more adaptive technology use and sleep-related behaviors.

By integrating SDT with behavioral substitution and self-regulatory frameworks, this study advances a statistically supported chain mediation architecture that clarifies how motivation quality is associated with downstream behavioral patterns. The findings underscore the dynamic and potentially reciprocal nature of these processes. Autonomous motivation is associated with greater physical activity engagement, and sustained engagement may, in turn, reinforce the internalization of motivational regulation.

From an applied perspective, this pattern suggests that interventions targeting the enhancement of autonomous motivation for physical activity may yield cumulative benefits. Initial enhancements in motivation may be linked to improved exercise adherence, which is associated with more favorable psychological and behavioral outcomes, thereby potentially reinforcing motivational regulation within a broader ecological context.

## Data Availability

The data and materials are not publicly available due to participant privacy but are available from the corresponding author upon reasonable request by qualified researchers.
